# Hyperbaric Oxygen Therapy Enhanced Circulating Levels of Endothelial Progenitor Cells and Angiogenesis Biomarkers, Blood Flow, in Ischemic Areas in Patients with Peripheral Arterial Occlusive Disease

**DOI:** 10.3390/jcm7120548

**Published:** 2018-12-14

**Authors:** Pao-Yuan Lin, Pei-Hsun Sung, Sheng-Ying Chung, Shan-Ling Hsu, Wen-Jung Chung, Jiunn-Jye Sheu, Shu-Kai Hsueh, Kuan-Hung Chen, Re-Wen Wu, Hon-Kan Yip

**Affiliations:** 1 Department of Plastic and Reconstructive Surgery, Kaohsiung Chang Gung Memorial Hospital and Chang Gung University College of Medicine, Kaohsiung 83301, Taiwan; Paoyuan9219@gmail.com; 2 Division of Cardiology, Department of Internal Medicine, Kaohsiung Chang Gung Memorial Hospital and Chang Gung University College of Medicine, Kaohsiung 83301, Taiwan; e12281@cgmh.org.tw (P.-H.S.); miosheny@cgmh.org.tw (S.-Y.C.); rylchen.msu@gmail.com (W.-J.C.); tang@cgmh.org.tw (S.-K.H.); 3 Department of Orthopedics, Kaohsiung Chang Gung Memorial Hospital and Chang Gung University College of Medicine, Kaohsiung 83301, Taiwan; hsishanlin@yahoo.com.tw; 4 Division of thoracic and Cardiovascular Surgery, Department of Surgery, Kaohsiung Chang Gung Memorial Hospital and Chang Gung University College of Medicine, Kaohsiung 83301, Taiwan; jiunnjye@cgmh.org.tw; 5 Department of Anesthesiology, Kaohsiung Chang Gung Memorial Hospital and Chang Gung University College of Medicine, Kaohsiung 83301, Taiwan; amigo6463@cgmh.org.tw; 6 Center for Shockwave Medicine and Tissue Engineering, Kaohsiung Chang Gung Memorial Hospital and Chang Gung University College of Medicine, Kaohsiung 83301, Taiwan; 7 Institute for Translational Research in Biomedicine, Kaohsiung Chang Gung Memorial Hospital, Kaohsiung 83301, Taiwan; 8 Department of Medical Research, China Medical University Hospital, China Medical University, Taichung 40402, Taiwan; 9 Department of Nursing, Asia University, Taichung 41354, Taiwan

**Keywords:** peripheral arterial occlusive disease, hyperbaric oxygen, circulating levels of endothelial progenitor cells and soluble angiogenesis biomarkers, blood flow

## Abstract

Background: This study tested the hypothesis that hyperbaric oxygen (HBO) therapy enhanced the circulating levels of endothelial progenitor cells (EPCs), soluble angiogenesis factors, and blood flow in ischemic areas in patients with peripheral arterial occlusive disease (PAOD). Methods: In total, 57 consecutive patients with PAOD undergoing the HBO therapy (3 atmospheres (atm) for 2 h each time) were prospectively enrolled into the present study. Venous blood sampling was performed to assess the circulating levels of EPCs and soluble angiogenesis factors prior to and during five sessions of HBO therapy. Additionally, skin perfusion pressure (SPP), an indicator of blood flow in ischemic areas, was measured by moorVMS-PRES. Results: The results demonstrated that the circulating levels of EPCs (cluster of differentiation (CD)34^+^/CD133^+^/CD45^dim^, CD31^+^/CD133^+^/CD45^dim^, CD34^+^) and soluble angiogenesis factors—vascular endothelial growth factor/stromal cell-derived factor 1/hepatocyte growth factor/fibroblast growth factor (VEGF/SDF-1α/HGF/FGF) were significantly increased post-HBO therapy as compared to pre-HBO therapy (all *p* < 0.01). Additionally, Matrigel assay showed that the angiogenesis was significantly increased in post-HBO therapy as compared to prior to therapy (*p* < 0.001). Furthermore, SPP was significantly increased in the ischemic area (i.e., plantar foot and mean SPP of the ischemic foot) in post-HBO therapy as compared to pre-HBO therapy (all *p* < 0.01). Importantly, the HBO therapy did appear to result in complications, and all the patients were uneventfully discharged without amputation. Conclusions: HBO therapy augmented circulating levels of EPCs and angiogenesis factors, and improved the blood flow in the ischemic area.

## 1. Introduction

Arterial atherosclerotic occlusive syndrome (AAOS) remains the leading cause of mortality and morbidity worldwide. It is well recognized that peripheral arterial occlusive disease (PAOD) is not only one of the leading contributors to AAOS, but also commonly occurs in those of advanced age and in diabetic patients [1,2,3,4]. PAOD is a manifestation of atherosclerosis in the lower extremities [1,2,3,4]. The true prevalence of PAOD in the general population is rather difficult to identify [5]. The recent data available from a screening trial of Danish men aged 65 to 74 years reported that the prevalence of PAOD was 11% when PAOD was defined as the presence of an ankle–brachial index (ABI) of less than 0.9 or greater than 1.4 [5]. Another recent report [6] found that over 5 years of follow-up approximately 7% of patients with asymptomatic PAOD developed intermittent claudication and approximately 21% of patients with intermittent claudication progressed to critical limb ischemia, with a 5-year cumulative incidence of cardiovascular mortality of 9% [6]. Therefore, patients with PAOD not only are at increased risk for cardiovascular disease (CVD) events, but also have higher mortality rates of cerebrovascular disease and critical limb ischemia (CLI) owing to systemic atherosclerosis [7].

PAOD can jeopardize walking, especially in severe cases, and can cause tissue loss, infection, and even amputation. Treatment for CLI is still a formidable challenge to clinicians [8]. Without appropriate treatment, the one-year mortality rate for CLI has been estimated to be as high as 25% [9]. Failure to treat CLI may also lead to limb loss and high costs of patient care following amputation. For those CLI patients who are not candidates for surgical or endovascular treatment and those with failure of revascularization or bypass occlusion, clinical outcomes remain gloomy. Therefore, the development of an alternative strategy with safety and efficacy for the treatment of CLI patients who are refractory to conventional therapy is of paramount importance.

Hyperbaric oxygen (HBO) therapy is a traditional therapy for patients with ischemic PAOD [10,11,12]. Accordingly, this therapy is a standard accessory method that has been regularly used with PAOD patients in Kaohsiung Chang Gung Memorial Hospital for over a decade. The underlying mechanism of HBO therapy involved in improving ischemic PAOD has been proposed to be mainly through an increase of vascular wall permeability and production of hypoxia-inducible factor-1α (HIF-1α) and stromal cell-derived factor (SDF)-1α, whicih enhance the angiogenesis and blood flow in the ischemic area [12]. It is well known that circulating endothelial progenitor cells (EPCs) play a crucial role in angiogenesis [13,14,15]. Surprisingly, whether HBO therapy would enhance the circulating level of EPCs in PAOD patients has not yet been reported. Thus, the aim of our study is to investigate effect of HBO therapy on enhancing circulating EPCs and blood flow in ischemic regions in patients with PAOD.

## 2. Methods

### 2.1. Ethics

In our hospital, HBO therapy is routinely applied to patients with intractable wound healing processes caused by ischemia, diabetic foot, ischemic stroke, and other causal etiology, as well as to PAOD patients with poor response to conventional therapy. From October 2016 to July 2018, a total of 57 consecutive patients who had PAOD with a 100% wound ulcer and a poor would healing situation who were eligible for HBO therapy were prospectively enrolled into the study. The study protocol was approved by the institutional review board (201600273B0) and conducted at Kaohsiung Chang Gung Memorial Hospital, a tertiary referral center. Written informed consent was obtained from all study participants prior to enrollment. 

### 2.2. Inclusion and Exclusion Criteria 

All patients aged between 20 and 80 years who had PAOD were eligible for the HBO therapy. On the other hand, patients with history of the following conditions were excluded from the study: surgery, trauma, or myocardial infarction within the preceding 3 months, liver cirrhosis, hematology disorders, malignancy, febrile disorders, acute or chronic inflammatory disease at study enrollment, congestive heart failure (New York Heart Association (NYHA) Functional class IV), age <20 or >80 years, diabetic retinopathy, or pregnancy. Additionally, those who were candidates for percutaneous transluminal angioplasty/stenting or bypass surgery and those with CLI were also excluded from present study.

### 2.3. Flow Cytometry for Assessing Circulating EPC Levels and ELISA for Measuring Soluble Angiogenesis Factors 

The procedure and protocol were based on our previous report [14]. In detail, blood samples were consecutively drawn at 08:00 h prior to and after the third and fifth session of HBO therapy in each patient. EPCs (CD34^+^/KDR^+^/CD45^dim^, CD34^+^/CD133^+^/CD45^dim^, CD31^+^/CD133^+^/CD45^dim^, CD34^+^/CD133^+^/KDR^+^, CD34^+^) in circulatory blood were identified by flow cytometry using double staining, as depicted in our recent report [14] through a fluorescence-activated cell sorter (FACSCalibur^TM^ system; Beckman Coulter Inc., Brea, CA, USA). Each analysis included 300,000 cells per sample. 

On the other hand, circulating levels of vascular endothelial growth factor (VEGF), hypoxia-inducible factor (HIF)-1α, hepatocyte growth factor (HGF), and stromal cell-derived growth factor (SDF)-1α, four indicators of soluble angiogenesis biomarkers, were measured by duplicated determination with a commercially available ELISA method (R&D Systems, Minneapolis, MN, USA). Intra-observer variability of the measurements was also derived, and the mean intra-assay coefficients of variance were all <4.0%. Additionally, other laboratory parameters were measured by following standard procedures in the Department of Clinical Biochemistry and Pathology of our hospital.

### 2.4. Culture of Endothelial Progenitor Cells from Peripheral Blood-Derived Mononuclear Cells 

For the purpose of ECP culture, blood samplings were collected from randomized 10 patients in the present study. After the peripheral blood sample was collected from venous puncture, the isolated mononuclear cells were cultured in a 100-mm diameter dish with 10 mL of Dulbecco’s modification of Eagle medium culture medium containing 10% fetal bovine serum. By day 21 of culturing, abundant peripheral blood-derived EPCs were obtained from each blood sampling. 

### 2.5. Matrigel Assay for Angiogenesis

To elucidate the therapeutic effect of HBO on in vitro angiogenesis, Matrigel assay was performed in the present study. The protocol and procedure of assessment of angiogenesis were based on our recent report with some modifications [15]. In brief, circulatory mononuclear cell-derived EPCs were placed in 48-well plates at 3.0 × 10^4^ cells/well in 100 µL serum-free M199 culture medium mixed with 100 µL cold Matrigel (Chemicon International, Inc., Temecula, CA, USA) for 3 h and incubated in 5% CO2 at 37 °C. Three random microscopic images (200×) were taken from each well to count cluster, tube and network formations, and the mean values were obtained. Both cumulative and mean tube lengths were calculated by Image-Pro Plus software (Media Cybernetics, Bethesda, MD, USA).

### 2.6. Procedure and Protocol of Hyperbaric Oxygen Therapy 

The HBO therapy was performed for the patients in a sealed multi-place chamber at a pressure of 2.5 atmospheres absolute (ATA). Air pressure was gradually increased from 1 ATA to 2.5 ATA over a 15-min duration. Oxygen of 100% medical grade was inhaled through a plastic facemask for 25 min followed by a 5-min break for a total of 90 min per treatment. Air pressure was then decompressed from 2.5 ATA down to 1.0 ATA within 15 min to complete the treatment. HBO was performed daily, five times a week, for a total of 10–15 treatments. 

All patients received HBO therapy were safe without any complication irrespective of coming from hospitalization or outpatient department.

### 2.7. Statistical Analysis

Quantitative data are expressed as mean ± SD. Statistical analysis was adequately performed by ANOVA followed by Bonferroni multiple comparison post hoc test. Statistical analysis was performed using SAS statistical software for Windows version 8.2 (SAS institute, Cary, NC, USA). A probability value of less than 0.05 was considered statistically significant.

## 3. Results

### 3.1. Baseline Characteristics of the 57 Study Patients (Table 1)

The mean age of participants was 60.9 years old and more than 54.0% were of male gender. The highest incidence of coronary artery disease (CAD) risk factors was hypertension (i.e., 54.4%), followed by diabetes mellitus. The frequency of chronic kidney disease was 19% and the laboratory findings showed that the data of hemogram was within the normal range.

### 3.2. Comparison of Circulatory EPC Levels and Skin Perfusion Pressure (SPP) of Ischemic Foot before and after HBO Therapy (Table 2)

The circulating levels of CD34^+^/KDR^+^/CD45^dim^ and CD34^+^/CD133^+^/KDR^+^, two kinds of EPC surface markers, did not differ between pre-HBO and post-HBO therapy. However, the circulating levels of CD34^+^/CD133^+^/CD45^dim^, CD31^+^/CD133^+^/CD45^dim^, and CD34^+^, another three indicators of EPC surface markers, were significantly higher in post-HBO therapy as compared to pre-HBO therapy among the study patients. Additionally, microcirculatory assessment (moorVMS-PRES, Moor Instruments, Wilmington, DE, USA) demonstrated that the SPP in the plantar foot area and the mean SPP of the whole ischemic foot area, two indicators of microcirculatory blood flow, were significantly increased in post-HBO as compared to pre-HBO therapy among the study patients.

### 3.3. Circulating Levels of Inflammatory and Soluble Angiogenesis Biomarkers (Figure 1)

The result of ELISA demonstrated that the circulatory level of interleukin (IL)-6, an indicator of inflammation, was significantly reduced in the first administration of HBO therapy and further significantly reduced in the second administration of HBO therapy as compared to pre-HBO therapy. On the other hand, the circulating levels of VEGF, SDF-1α, and HGF, three indices of soluble angiogenesis factors, exhibited an opposite pattern of IL-6 among the three-time intervals. However, the circulating level of HIF-α, another indicator of soluble angiogenesis factor, was significantly and progressively reduced after HBO therapy, suggesting in intrinsic response to the improvement of ischemia after HBO therapy.

### 3.4. Matrigel Assay for Determinant Angiogenesis (Figure 2)

The Matrigel assay (i.e., in vitro study) demonstrated that the angiogenesis capacity was significantly enhanced in post-HBO as compared to pre-HBO therapy.

## 4. Discussion

This study, which investigated the therapeutic impact of HBO therapy with respect to circulatory angiogenesis factors and blood flow in ischemic areas, yielded several striking implications. First, the circulating level of EPCs was markedly enhanced in patients after the fifth administration of HBO therapy. Second, consistent with the EPC level, the circulatory angiogenesis factors were also substantially increased after HBO therapy, whereas the inflammatory biomarker was notably ameliorated after HBO therapy. The in vitro study (i.e., Matrigel assay) exhibited that the angiogenesis ability was also remarkably enhanced after HBO therapy.

The underlying mechanisms of HBO therapy for improving ischemic organ dysfunction have been established mainly through: (1) an increased oxygen pressure (i.e., transient hyperoxia) in ischemic area; (2) an increase of vascular wall permeability; and (3) increased generation of HIF-1α and SDF-1α, that, in turn, enhanced the angiogenesis and blood flow in the ischemic area [10]. Intriguingly, when we reviewed the literature, we found that there is yet no any data to address the impact of HBO therapy on regulating the circulating levels of angiogenesis factors (i.e., EPCs and soluble angiogenesis factors) in PAOD patients who are not candidates for intervention (including percutaneous transluminal angioplasty/stenting or bypass surgery) but are alternatively suitable for HBO therapy. Accordingly, to the best of our knowledge, this is the first study investigating the therapeutic impact of HBO on circulating level of EPCs in PAOD patients. The most important finding in the present study was that the EPC level was remarkably increased after the HBO therapy. Abundant data have shown that EPCs play an essential role on endothelial repair and angiogenesis in ischemic areas for restoring the blood flow in ischemic organs [13,14,15]. Therefore, our finding, besides being consistent with the findings from the previous studies [13,14,15], could (at least in part) explain why the blood flow increased in the PAOD ischemic area. Of particular importance is that these circulatory biomarkers that have not been keenly discussed with respect to PAOD patients could be considered as fundamental parameters predictive of prognostic outcomes in clinical settings of PAOD patients after receiving HBO therapy.

The link between increased circulating levels of soluble angiogenesis biomarkers and enhancement of microvasculature/neovascularization has been keenly investigated by previous studies [13,16]. Additionally, other previous studies have also identified that HBO therapy increased circuiting levels of soluble angiogenesis factors [12], augmented the wound healing process [17,18], and ameliorated the ischemia in cardiovascular disease [19]. A principal finding in the present study was that the circulatory levels of angiogenesis biomarkers were significantly increased after HBO therapy. In this way, our finding, in addition to corroborating the findings of the previous studies [12,13,16,17,18,19], once again explained why the microcirculatory blood flow was notably increased after HBO therapy. Interestingly, the result of the present study also found that the circulating levels of inflammatory biomarkers were remarkably suppressed by HBO therapy, highlighting that HBO therapy would ameliorate the inflammation that may be due to a reduction in tissue ischemia-related inflammatory stimulation. 

The Matrigel assay technique was utilized to prove the direct effect of HBO on cellular angiogenesis in the present study. As we expected, the Matrigel assay demonstrated that the angiogenesis ability was markedly enhanced in post-HBO therapy as compared to pre-HBO therapy. This result, a ground-breaking pioneering finding could, once again, explain why the blood flow was improved in the ischemic area after HBO therapy.

### Study Limitations

This study has several limitations. First, those PAOD patients without HBO therapy were not enrolled into the study as a control group. Thus, we do not provide further information regarding the long-term outcomes for comparison after HBO therapy. Second, imaging studies such as arterial angiographic study, computerized tomographic angiographic study, or magnetic resonance imaging (MRI) study were not performed in the present study. We, therefore, do not provide the correlation between angiogenesis in ischemic areas and the circulating levels of angiogenesis factors, or a Matrigel assay of angiogenesis.

## 5. Conclusions

In conclusion, the improvement of blood flow in ischemic areas through HBO therapy might be mainly attributed to the enhancement of circulating molecular–cellular angiogenesis factors.

## Figures and Tables

**Figure 1:**
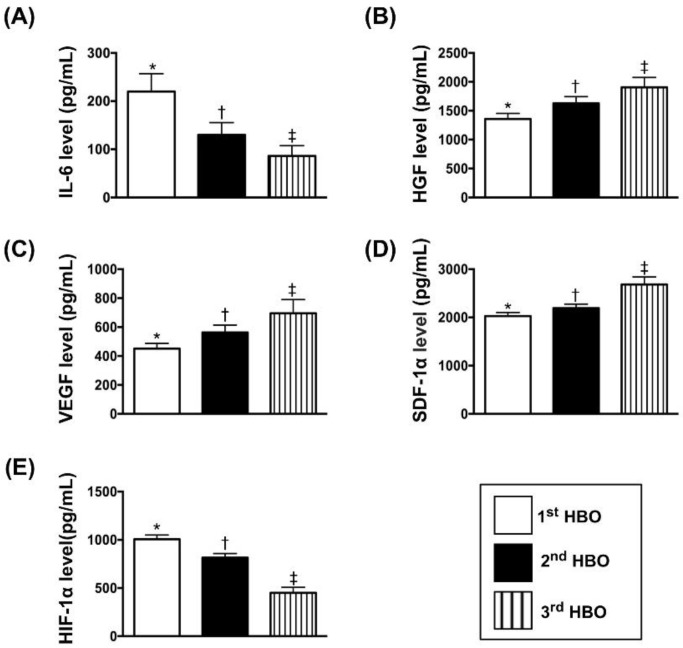
Time courses of circulating levels of inflammatory and soluble angiogenesis biomarkers. (**A**) Circulating level of interleukin (IL)-6, *, vs. other groups with different symbols (†, ‡), *p* < 0.0001. (**B**) Circulating level of hepatocyte growth factor (HGF), *, vs. other groups with different symbols (†, ‡), *p* < 0.0001. (**C**) Circulating level of vascular endothelial growth factor (VEGF), *, vs. other groups with different symbols (†, ‡), *p* < 0.0001. (**D**) Circulating levels of stromal cell-derived growth factor (SDF)-1α, *, vs. other groups with different symbols (†, ‡), *p* < 0.0001. (**E**) Circulating level of hypoxia-induced factor (HIF)-1α, *, vs. other groups with different symbols (†, ‡), *p* < 0.0001. All statistical analyses were performed by one-way ANOVA, followed by Bonferroni multiple comparison post hoc test (*n* = 57 for each time interval). Symbols (*, †, ‡) indicate significance at the 0.05 level. “1st” indicates the blood sample was drawn prior to HBO therapy; “2nd” and “3rd” indicate the blood samples were drawn after the third and fifth sessions of HBO therapy, respectively.

**Figure 2:**
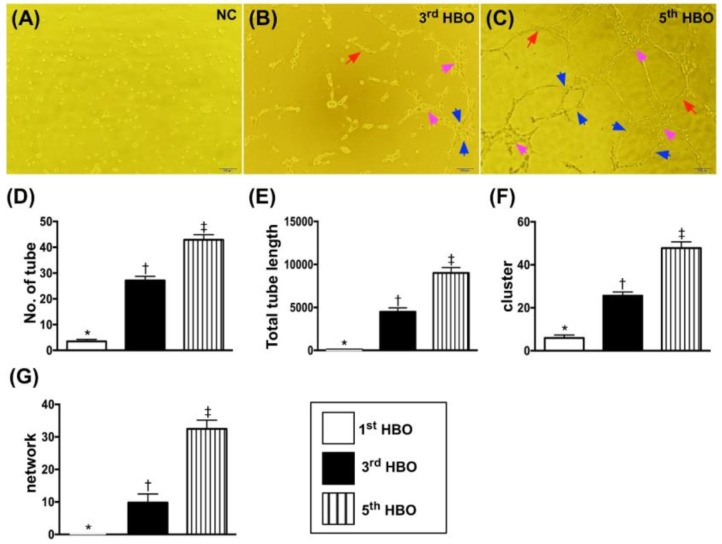
Matrigel assay for angiogenesis formation. (**A**–**C**) Illustrates the angiogenesis features of one patient prior to (**A**) and after the third (**B**) and fifth (**C**) sessions of HBO therapy, respectively. The angiogenesis was notably increased after the third session of HBO therapy and more notably increased after the fifth session of HBO therapy. The tubular (red arrows), cluster (pink arrows), and network (blue color) formations. (**D**) Number of tubule formations, *, vs. other groups with different symbols (†, ‡), *p* < 0.0001. (**E**) Tubule length, *, vs. other groups with different symbols (†, ‡), *p* < 0.0001. (**F**) Number of cluster formations, *, vs. other groups with different symbols (†, ‡), *p* < 0.0001. (**G**) Number of network formations, *, vs. other groups with different symbols (†, ‡), *p* < 0.0001. All statistical analyses were performed by one-way ANOVA, followed by Bonferroni multiple comparison post hoc test (*n* = 10 patients). Symbols (*, †, ‡) indicate significance at the 0.05 level. “1st” indicates the blood sample was drawn prior to HBO therapy; “2nd” and “3rd” indicate the blood samples were drawn after the third and fifth sessions of HBO therapy, respectively.

**Table 1:** Baseline characteristics among the patients receiving HBO therapy.

Variables	Total (*n* = 57)
Age (years)	60.9 ± 14.1
Male gender	54.4% (31)
Current smoker	8.8% (5)
Hypertension	54.4% (31)
Diabetes	52.6% (30)
Dyslipidemia	41.8% (1)
Coronary artery disease	8.8% (5)
Chronic kidney disease	19.3% (11)
End-stage renal disease	14% (8)
Old myocardial infarction	1.8% (1)
Old ischemic stroke	7.0% (4)
Antibiotic therapy	31.6% (18)
Times of HBO therapy	10.0 ± 4.0
White blood count (1000/μL)	8.9 ± 3.5
Red blood count (10^6^/μL)	4.1 ± 0.7
Hemoglobin (g/dL)	11.5 ± 2.2
Serum creatinine (mg/dL)	2.3 ± 3.5
Blood urea nitrogen (mg/dL)	23.3 ± 19.1

Abbreviations: HBO = hyperbaric oxygen; Data are expressed as mean ± standard deviation (SD) or % (*n*).

**Table 2:** Change in circulatory EPCs and SPP of the ischemic foot before and after HBO therapy.

Variables	Pre-HBO	Post-HBO	*P*-Value
Flow cytometry (%) prior to and at 5th HBO therapy			
CD34^+^/KDR^+^/CD45^dim^	17.3 ± 15.3	22.1 ± 18.0	0.145
CD34^+^/CD133^+^/CD45^dim^	13.8 ± 9.8	20.2 ± 13.4	<0.001
CD31^+^/CD133^+^/CD45^dim^	12.9 ± 11.7	19.1 ± 13.9	0.012
CD34^+^/CD133^+^/KDR^+^	2.9 ± 2.1	5.3 ± 8.7	0.105
CD34^+^	82.9 ± 85.7	115.9 ± 99.1	0.001
Microcirculatory assessment (SPP, mmHg) 2 weeks after HBO therapy			
Dorsal foot	67.6 ± 24.4	75.9 ± 25.7	0.088
Plantar foot	57.2 ± 20.9	79.9 ± 30.8	0.007
Lateral foot	72.1 ± 26.5	79.2 ± 19.3	0.535
Medial foot	57.5 ± 24.6	78.9 ± 43.1	0.196
Mean SPP of the ischemic foot	62.1 ± 18.1	75.4 ± 20.3	0.003

Abbreviations: EPCs = endothelial progenitor cells; SPP = skin perfusion pressure; HBO = hyperbaric oxygen. Data are expressed as mean ± SD or % (*n*).
